# TRPV6 plays a new role in predicting survival of patients with esophageal squamous cell carcinoma

**DOI:** 10.1186/s13000-016-0457-7

**Published:** 2016-01-27

**Authors:** Shui-Shen Zhang, Xuan Xie, Jing Wen, Kong-Jia Luo, Qian-wen Liu, Hong Yang, Yi Hu, Jian-Hua Fu

**Affiliations:** State Key Laboratory of Oncology in South China, Sun Yat-Sen University Cancer Center, Guangzhou, People’s Republic of China; Guangdong Esophageal Cancer Research Institute, Guangzhou, People’s Republic of China; Department of Thoracic Oncology, Sun Yat-Sen University Cancer Center, 651 Dongfeng Road East, Guangzhou, 510060 People’s Republic of China; Department of Thoracic Surgery, the First Affiliated Hospital, Sun Yat-Sen University, Guangzhou, People’s Republic of China; Department of Thoracic Surgery, Sun Yat-Sen Memorial Hospital, Sun Yat-Sen University, Guangzhou, People’s Republic of China

**Keywords:** TRPV6, Esophageal squamous cell carcinoma, Prognosis; survival analysis, Tumor markers

## Abstract

**Background:**

TRPV6 is over-expressed and promotes the proliferation and invasion in many cancers. The association between the expression of TRPV6 and clinical outcome in esophageal squamous cell carcinoma (ESCC) has not been studied yet. We aim to elucidate the role of TRPV6 in predicting prognosis of patients with ESCC.

**Methods:**

In the retrospective study, mRNA level of TRPV6 was examined in patients (*N* = 174) from Sun Yat-sen University Cancer Center (mRNA cohort) and protein level of TRPV6 was examined in patients (*N* = 218) from Linzhou Cancer Hospital (protein cohort). Statistical analysis was performed to test the clinical and prognostic significance of TRPV6.

**Results:**

TRPV6 was down-regulated in ESCC tissues and cell lines. Patients with downregulation of TRPV6 trended to have a higher rate of advanced pT stage in both mRNA cohort (*P* = 0.089) and protein cohort (*P* = 0.073), though not statistically significant. No significant association was observed between TRPV6 expression and disease-specific survival (DSS) in both two cohorts. However, stratified survival analysis based on the gender showed that in mRNA cohort, downregulation of TRPV6 was associated with an unfavorable 3-year DSS in patients with male (47.3 % vs 63.6 %, *P* = 0.027) and with favorable 3-year DSS in patients with female (66.7 % vs 43.0 %, *P* = 0.031). The result was confirmed in protein cohort. Male patients with downregulation of TRPV6 had a poor 3-year DSS (20.0 % vs 57.1 %,*P* < 0.001) while female counterparts showed an enhanced 3-year DSS (56.1 % vs 28.6 %, *P* = 0.005).

**Conclusion:**

TRPV6 is down-regulated in ESCC. As a predictive biomarker, TRPV6 plays a Janus-like role in predicting survival of male and female ESCC patients.

## Background

Esophageal cancer is one of the most aggressive malignancies worldwide, with more than 480,000 new cases and 400,000 deaths annually. In China, esophageal squamous cell carcinoma (ESCC) ranks 4^th^ among most common causes of death and remains a major burden for public health [1]. Despite advances of surgical techniques and incorporation of new therapeutic approaches, ESCC is still a highly devastating disease with poor prognosis, 5-year overall survival at less than 40 % [2, 3]. Current researches have been focusing on prognostic predictor of ESCC in order to actively adapt therapies for the high-risk subpopulations.

Transient receptor potential (TRP) channels are a large family of nonselective ion channel which play a key role in a variety of physiological function [4]. More importantly, most of these channels have been proved to be associated with several diseases including cancers [5]. Several channels were identified to play crucial roles in the progression and prognosis of cancers [6–10].

TRPV6 (transient receptor potential vanilloid 6), is one of the most highly calcium selective channel among all TRP channels. Compared to other TRP channels, TRPV6 is characterized by its high selectivity for calcium and its active role in Ca^2+^-related intracellular pathway [11, 12]. Several studies have demonstrated that TRPV6 is over-expressed and promotes the proliferation and invasion in many cancers, such as prostate cancer [9, 13], breast [8, 14, 15] and colon cancer [16]. Lehen’kyi et al. [13] showed that TRPV6 is positively involved in regulation of proliferation in prostate cancer cell line LNCaP. Genetic silencing of TRPV6 leads to decreased proliferation rate, cell accumulation into S-phase of the cell cycle, and proliferating cell nuclear antigen (PCNA) expression. The study also revealed that Ca^2+^ uptake into LNCaP cells is mediated by TRPV6, with the subsequent downstream activation of nuclear factor of activated T cells (NFAT). High expression of TRPV6 mRNA in prostate cancer is closely associated with the elevated degree of aggressiveness of the cancer, assessed by the Gleeson score (grading of the pathological stage) and extraprostatic extension [9]. Besides, TRPV6 is mainly over-expressed in the invasive breast cancer cells and selective silencing of TRPV6 inhibits migration and invasion of breast cancer [15]. Patients with high expression of TRPV6 exhibits a worse survival when compared to those with low or intermediate TRPV6 expression in breast cancer [14]. These observations suggested that TRPV6 has oncogenic potential and could be used as a biomarker to predict the clinical outcome in most cancers. On the other hand, however, some studies indicated that TRPV6 might play antitumour role in some cancer types, such as colon cancer [17, 18]. Curcumin, which is a ligand of 1,25-vitamin D3 receptor that up-regulated TRPV6 in vivo, may play a role similar to that of 1,25-vitamin D3 in promoting calcium uptake as part of the protective effect against colon cancer [17]. Other study showed that TRPV6 can mediate capsaicin-induced apoptosis in gastric cells [18]. Studies thus far have shown that TRPV6 plays very complicated roles among different cancer types. Nevertheless, the expression pattern of TRPV6 in ESCC and its value in predicting the survival of patients has not been elucidated yet. Therefore, we aim to clarify the TRPV6 expression in primary ESCC and analyze the relation with prognosis in ESCC patients.

## Methods

### Patients and tissues specimens

In our retrospective study, 174 consecutive patients of ESCCs tissues and 45 patients of paired adjacent nontumor tissues were collected immediately after surgery resection at Sun Yat-sen University Cancer Center from March 2002 to October 2008 [10, 19]. As a validation, a total of 300 patients of formalin-fixed, paraffin-embedded ESCC tumor specimens and the corresponding normal epithelia were selected from another institute, Linzhou Cancer Hospital [20]. The cases selected were based on criteria described previously [10]. Briefly, inclusion criteria were as follows: histologic proof of thoracic ESCC, complete surgical resection (R0), and complete follow-up data. The exclusion criteria were: received neoadjuvant or adjuvant treatment, history of other malignancy, death in perioperative period, and cervical lymph nodes metastases. The preoperative workup was evaluated by endoscopy with biopsy and histologic examination, barium esophagography to confirm tumour location, thoracic and abdominal computed tomography (CT), and cervical ultrasonography (with biopsy if indicated) if cervical lymph node metastasis was suspected. Histological differentiation, pT category (depth of tumor invasion), and pN category (lymph node metastasis) were determined by pathologic examination. All patients gave written informed consent before operation. The study was approved by the Ethics Committee of the Cancer Center of Sun Yat-sen University and Linzhou Cancer Hospital.

### Cell culture

The ESCC cell lines, including KYSE30, KYSE140, KYSE180, KYSE 410, KYSE510, KYSE520, HKESC1, CE81T, EC109 and EC9706were all kindly provided by Prof. Xinyuan Guan from Hong Kong University, and maintained in Dulbecco’s Modified Eagle Medium supplemented with 10 % fetal bovine serum and 10 % penicillin-streptomycin at 37 °C in a humidified incubator containing 5 % CO_2_.

### Quantitative Real-time Polymerase Chain Reaction (qRT-PCR) assays

The fresh tumorous and non-tumorous samples were taken from regions which macroscopically judged to be neoplastic and normal, respectively. Both of them were immediately stored at dry ice after resection and then frozen at −80 °C. Total RNA of the specimens and exponentially growing cells were extracted by TRIzol reagent (Invitrogen) according to manufacturer’s instruction. Each cDNA was synthesized from 1 μg of total RNA using RevertAid First Strand cDNA Synthesis Kit (Thermo Scientific), and stored at −80 °C. cDNA was subjected to quantitative Real-Time PCR (qRT-PCR) for TRPV6. GAPDH was used as internal control. The primers used were as follows: TRPV6, forward primer 5'-CGTGTTCTCACTTCGCTTCCTGG-3' and reverse primer 5'-TGGCGTTCATGCTACTCCTCTTTC-3' [NCBI: NM_018646.5]; GAPDH, forward primer 5'-ACTTCAACAGCGACACCCACTC-3' and reverse primer 5'-TACCAGGAAATGAGCTTGACAAAG-3' [NCBI: NM_001256799.1]. qRT-PCR was done using the Power SYBR Green PCR Master Mix(Applied Bio systems) and LightCycler480 384-well PCR system (Roche Diagnostics). An initial denaturing step at 95 °C for 10 min and 40 cycles at 95 °C for 10 s, 60 °C for 20 s, and 72 °Cfor 30 s. The assays were done in triplicate and values were normalized using the internal control. Quantitative data were exported and TRPV6 expression was normalized by internal control. PCR products were subjected to dissociation curve analysis to exclude amplification of nonspecific products. The value of relative expression was calculated using the 2^-△△Ct^ method. △△Ct(sample) = △Ct(sample)- △Ct(calibrator), △Ct(sample) = Ct(sample) of TRPV6-Ct(sample) of GAPDH; △Ct(calibrator) = Ct(calibrator) of TRPV6-Ct(calibrator) of GAPDH; Calibrator was defined as the pooled samples from 45 patients of adjacent nontumor tissues [10, 19].

### ESCC tissue microarray and immunohistochemical staining

The ESCC tissue microarray (TMA) with a total of 300 formalin-fixed, paraffin-embedded ESCC tumor specimens and the corresponding normal epithelia was kindly provided by Prof. Xinyuan Guan from State Key Laboratory of Oncology in Southern China, Sun Yat-Sen University Cancer Center. The ESCC tissue microarray was constructed as described previously [20, 21]. Briefly, TMA were constructed with a Beecher Instruments tissue microarrayer (Beecher Instruments, Sun Prairie, WI). Three targeted core samples with a 1-mm diameter of each specimen were punched and arrayed on a recipient paraffin block to construct the tissue microarray. For immunohistochemical (IHC) analysis, the slides were deparaffinized, rehydrated, and blocked by 10 % normal goat serum at room temperature for 30 min. The slides were then incubated with rabbit polyclonal antibody against TRPV6 (alomone labs) at a dilution of 1:50 at 4 °C overnight and subsequently incubated with biotinylated goat anti-rabbit immunoglobulin at a concentration of 1:100 for 30 min at 37 °C. A staining index (values 0–7) was calculated by adding the scores for the intensity of TRPV6-positive staining (negative, 0; weak, 1; moderate, 2; or strong, 3) and the percentage of TRPV6-positive cells (<25 %, 1; 25 %–50 %, 2, >50 %–75 %, 3; ≥ 75 %, 4 scores).

Two independent observers blinded to the clinicopathologic information performed the evaluation of TRPV6 expression. If the two observers conflicted with each other, a third independent observer was asked to determine the final result.

### Statistical methods

Receiver operative characteristic (ROC) curve generated by MedCalc 9.6.2.0 (MedCalc Software, Mariakerke, Belgium) was used to determine the cutoff value for TRPV6 mRNA expression that yielded the highest combined sensitivity and specificity with respect to distinguishing disease specific 5-year survivors from non-survivors [10, 22]. Statistical analysis was performed using the SPSS 16.0 for windows software system (SPSS Inc, Chicago, IL). Paired *t* test was adopted to compare the expression of TRPV6 mRNA in 45 pairs of primary ESCCs between tumor tissues and nontumor tissues. The correlation between TRPV6 expression and clinicopathologic parameters in mRNA cohort and protein cohort was analyzed by Chi-square test where applicable. Disease specific survival (DSS) was calculated from the time of surgery to the time of death from ESCC or last follow-up. To the time of last follow-up or death from disease other than ESCC, at which point, the data was censored. The prognostic value using expression of TRPV6 for predicting survival were calculated by the Kaplan-Meier method and analyzed by log-rank test. To determine independent factors that were significantly related to the prognosis, multivariate analysis was performed using Cox’s proportional hazards regression model with a forward stepwise procedure (the entry and removal probabilities were 0.05 and 0.10, respectively). A significant difference was declared if the *P* value from a two-tailed test was less than 0.050.

## Results

### TRPV6 was frequently down-regulated in ESCC

**Fig. 1:**
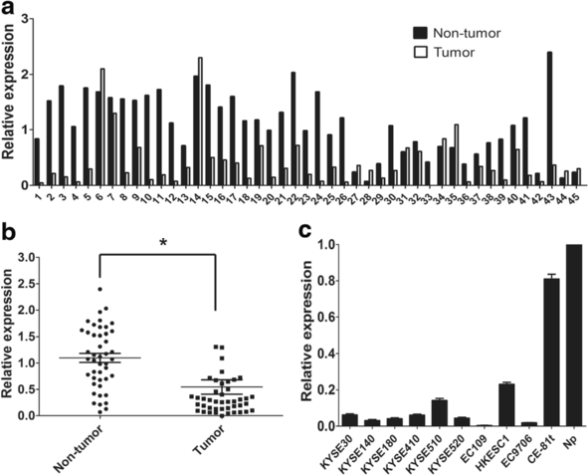
TRPV6 was down-regulated in esophageal squamous cell carcinoma. TRPV6 mRNA was markedly decreased in tumor tissues than that in paired adjacent non-tumor tissues (**a** and **b**). TRPV6 mRNA was down-regulated in ten esophageal squamous cell carcinoma cell lines when compared with pooled samples from 45 patients of adjacent non-tumor tissues (Np) (**c**)

The mRNA expression of TRPV6 was initially tested in 45 pairs of primary ESCC tumors and their adjacent nontumor tissues by qPCR. Downregulation of TRPV6 was detected in 32 of 45 (71.1 %) of ESCC tumors compared with paired adjacent nontumor tissues (defined as a 2-fold decrease of TRPV6 expression in tumors) (Fig. 1a). The relative expression level of TRPV6 was significantly down-regulated in tumor tissues compared with paired adjacent nontumor tissues (*P* < 0.001, Fig. 1b). The expression levels of TRPV6 in ten ESCC cell lines were also tested by qRT-PCR, and the results showed that TRPV6 was down-regulated in all of them, compared with pooled samples from 45 nontumor tissues (Np) (Fig. 1c).

**Fig. 2:**
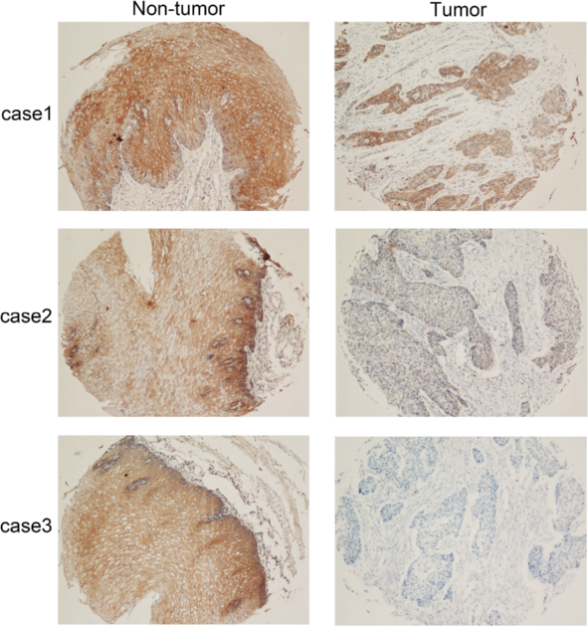
Representative of TRPV6 expression in three pairs of ESCC tumor and adjacent non-tumor tissue detected by immunostaining with anti-TRPV6 antibody (*brown*). (magnification: 100×)

TRPV6 expression in protein level was further studied in 300 primary ESCCs by IHC using a TMA. Informative IHC results were obtained from 244 pairs of ESCCs. Noninformative samples included lost samples, unrepresentative samples, and samples with too few tumor cells; such were not used in data complication. The staining index of TRPV6 in each informative nontumor tissue was equal or greater than 5; therefore, staining index 5–7 was counted as normal expression of TRPV6 whereas 0–4 was counted as downregulation of TRPV6. Using this designation, downregulation of TRPV6 was detected in 118 of 244 (48.4 %) informative ESCC tissues compared with their adjacent nontumor tissues (Fig. 2).

### Clinical and pathological data

174 patients with primary ESCCs from Sun Yat-sen University Cancer Center (mRNA cohort) and 218 patients with primary ESCCs from Linzhou Cancer Hospital (protein cohort) were recruited in this study. 26 out of 244 patients from Linzhou Cancer Hospital were excluded according to the exclusion criteria. In mRNA cohort, the optimal cutoff value of TRPV6 with the best discriminatory power was determined to be 0.207 based on the ROC curve. At this threshold of TRPV6, the sensitivity was 63.1 %, the specificity was 43.3 %. Then, TRPV6 mRNA expression in mRNA cohort was divided into two groups: downregulation (≤0.207, *n* = 66) and normal expression (>0.207, *n* = 108) group. Follow-up data were obtained from all patients, with a median survival of 39 months in mRNA cohort (range, 2–105months) and 24 months in protein cohort (range, 1–60months). According to the 7th edition AJCC staging system [23] and our demographic data, the clinicopathologic features were dichotomized for statistical analyses as shown in Table 1.

**Table 1:** The association between TRPV6 expression and clinicopathologic features in patients with ESCC

Characteristic	mRNA cohort			Protein cohort		
	Case	Downregulation (%)	*P* ^a^	Case	Downregulation (%)	*P* ^a^
Age			0.827			0.407
≤ 58	72	28(38.9)		113	58(51.3)	
> 58	102	38(37.3)		105	48 (45.7)	
Gender			0.298			0.093
Male	126	45(35.7)		121	65(53.7)	
Female	48	21(43.8)		97	41(42.3)	
Location			0.992			0.364
Upper	35	13(37.1)		49	20(40.8)	
Middle	94	36(38.3)		152	76(50.0)	
Lower	45	17(37.8)		17	10(58.8)	
Differentiation			0.133			0.398
Grade 1	41	16(39.0)		24	14(58.3)	
Grade 2	91	29(31.9)		147	67(45.6)	
Grade 3	42	21(50.0)		47	25(53.2)	
pT category			0.089			0.073
T1–2	43	15(34.4)		80	37(46.7)	
T3–4	131	64(48.8)		138	82(59.3)	
pN category			0.854			0.459
N0	96	37(38.5)		124	63(50.8)	
N1–3	78	29(37.2)		94	43(45.7)	
Pathological staging			0.827			0.365
I–II	101	39(38.6)		137	67(48.8)	
III	73	27(37.0)		81	42(52.2)	

^a^Chi-square test

### Clinical significance of TRPV6 expression in primary ESCC patients

The association between TRPV6 expression and clinicopathological features in mRNA cohort and protein cohort was summarized in Table 1. The result showed that patients with downregulation of TRPV6 trended to have a higher rate of advanced pT stage in both mRNA cohort (*P* = 0.089) and protein cohort (*P* = 0.073), but did not reach the significance. No significant association was observed between TRPV6 expression and age, gender, tumor location, histological differentiation, pN category and pathological stage either in mRNA cohort or protein cohort.

### Relation between TRPV6 expression and patients survival

**Fig. 3:**
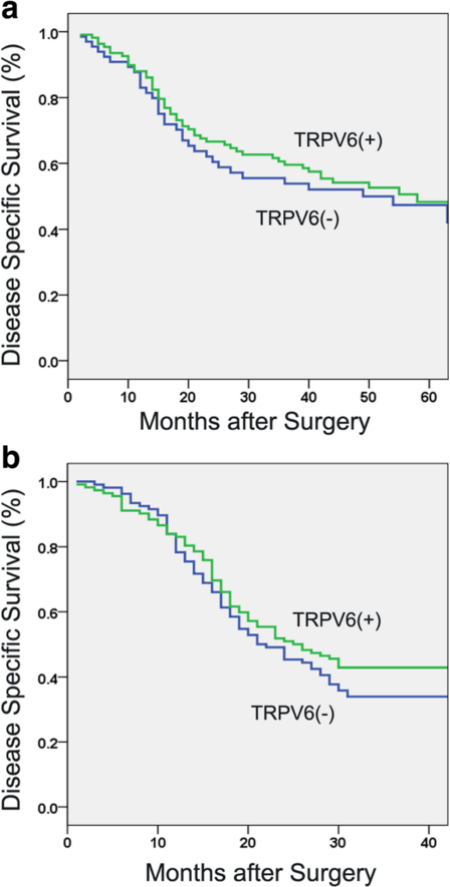
Kaplan-Meier curves showed that no significant association between TRPV6 expression and disease-specific survival of esophageal squamous cell carcinoma was noted either in mRNA cohort (**a**) or protein cohort (**b**)

**Fig. 4:**
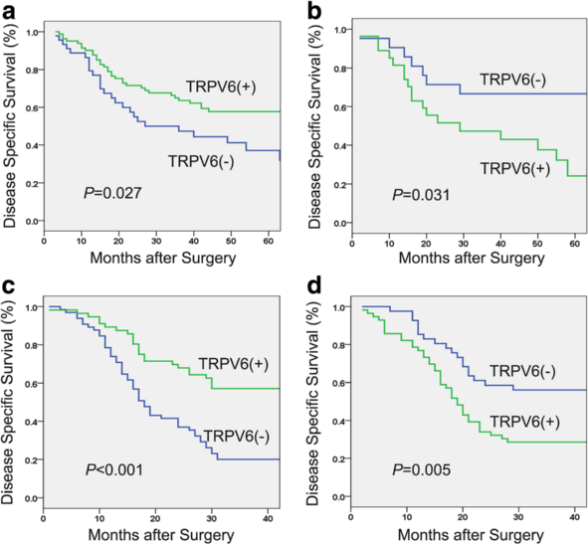
Univariate analysis showed that pT category (**a**), pN category (**b**), and pathological staging (**c**) were significantly associate disease specific survival of patients with esophageal squamous cell carcinoma in the mRNA cohort

**Fig. 5:**
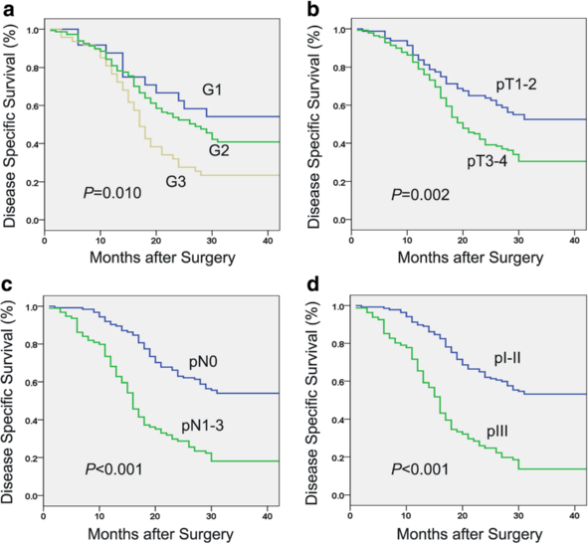
Univariate analysis showed that differentiation (**a**), pT category (**b**), pN category (**c**), and pathological staging (**d**) were significantly associate disease specific survival of patients with esophageal squamous cell carcinoma in the protein cohort

Kaplan-Meier analysis showed that patients with downregulation of TRPV6 trended to have a poorer 3-year disease specific survival (DSS) in mRNA cohort (53.8 % vs 59.6 %, *P* = 0.531, Fig. 3a) and in protein cohort (34.0 % vs 42.9 %, *P* = 0.244, Fig 3b), though not statistically significant. Univariate analysis showing in Table 2 demonstrated that pT category (*P* = 0.040 and 0.002, respectively, Figs. 4a, 5b), pN category (*P* < 0.001 and 0.001, respectively, Figs. 4b, 5c) and pathological staging (*P* < 0.001 and 0.001, respectively, Figs. 4c, 5d) were closely associated with 3-year DSS in both cohorts. Histological differentiation was found to be significantly associated with DSS only in protein cohort (*P* = 0.010, Fig. 5a) but not in mRNA cohort (*P* = 0.330). Further multivariate survival analysis showed that pN category (HR = 2.95, 95 % CI:1.89-4.62, *P* < 0.001) was an independent prognostic factor in mRNA cohort, pN category (HR = 2.93, 95 % CI:2.07-4.15, *P* < 0.001) and histological differentiation (HR = 1.58, 95 % CI:1.16-2.15, *P* = 0.004) were independent prognostic factors in protein cohort (Table 3).

**Table 2:** Univariate analysis of TRPV6 expression and clinicopathological factors for disease specific survival in patients with ESCC

Characteristic	mRNA cohort		Protein cohort	
	3-year DSS (%)	*P* ^a^	3-year DSS (%)	*P* ^a^
TRPV6 expression		0.531		0.244
Downregulation	53.8		34.0	
Normal expression	59.6		42.9	
Age		0.145		0.448
≤ 58	60.6		38.9	
> 58	55.3		38.1	
Gender		0.653		0.880
Male	58.1		40.2	
Female	55.9		37.2	
Location		0.545		0.428
Upper	64.4		30.6	
Middle	56.1		40.1	
Lower	55.2		47.1	
Differentiation		0.330		0.010
Grade 1	62.6		54.2	
Grade 2	60.7		40.8	
Grade 3	45.4		23.4	
pT category		0.040		0.002
T1–2	66.4		52.5	
T3–4	55.6		30.4	
pN category		<0.001		<0.001
N0	74.0		54.0	
N1–3	37.5		18.1	
Pathological staging		<0.001		<0.001
I–II	72.1		53.3	
III	37.5		13.6	

^a^Kaplan-Meier method (log-rank test)

DSS, disease specific survival

**Table 3:** Multivariate survival analysis^a^ for disease specific survival in patients with ESCC

Prognostic factor	mRNA cohort			Protein cohort		
	HR	95 % CI	*P*	HR	95 % CI	*P*
Differentiation	-	-	-	1.58	1.16-2.15	0.004
pT category	1.40	0.80-2.46	0.240	1.36	0.92-2.01	0.127
pN category	2.95	1.89-4.62	<0.001	2.93	2.07-4.15	<0.001

^a^Cox’s proportional hazards regression analysis (Forward stepwise)

HR, hazard ratio

95 % CI, 95 % confidence interval

-,unavailable

**Fig. 6:**
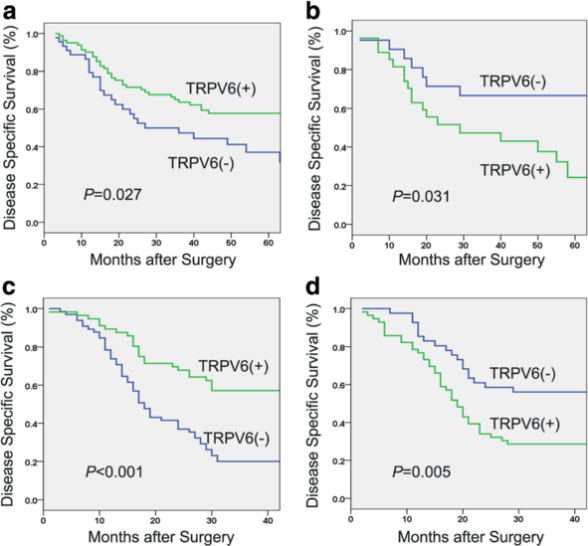
Two cohorts both showed that TRPV6 played a contradicting role in predicting survival of male and female patients. Downregulation of TRPV6 was associated with a poor 3-year disease specific survival in patients with male in mRNA (**a**) and protein cohort (**c**). Downregulation of TRPV6 was significantly associated with favorable 3-year disease specific survival in patients with female in mRNA (**b**) and protein cohort (**d**)

Due to the expression of TRPV6 regulated by sex steroid hormones, we conducted stratified survival analysis based on the gender. In mRNA cohort, downregulation of TRPV6 was significantly associated with an unfavorable 3-year DSS in patients with male (47.3 % vs 63.6 %, *P* = 0.027, Fig. 6a), while downregulation of TRPV6 was significantly related with a favorable 3-year DSS in patients with female (66.7 % vs 43.0 %, *P* = 0.031, Fig. 6b). More importantly, the result was validated in protein cohort. Male patients with downregulation of TRPV6 had a poor 3-year DSS (20.0 % vs 57.1 %,*P* < 0.001, Fig. 6c) and female patients with downregulation of TRPV6 had an increased 3-year DSS (56.1 % vs 28.6 %, *P* = 0.005, Fig. 6d).

## Discussion

In our study, we demonstrated that TRPV6 is generally down-regulated in ESCC tissues and cell lines. In both mRNA cohort and protein cohort, downregulation of TRPV6 is associated with unfavorable survival in male patients and with favorable survival in female patients. To the best of our knowledge, this is the first study to report the expression profile of TRPV6 in ESCC and its unique Janus-like role in predicting survival of male and female patients.

TRPV6 has been found to be expressed in normal epithelia from various organs, for instance, the linings of the gastrointestinal tract, kidney and so on [24, 25]. When compared with normal tissue or cells, the increased expression of TRPV6 at the mRNA and protein levels has been observed in prostate cancer [9, 13], breast cancer [8, 14, 15] and colon cancer [16]. Additionally, TRPV6 has been demonstrated to play a crucial role in promoting the progression of prostate cancer [13] and breast cancer [8]. One study found that TRPV6 mediates Ca^2+^ uptake into prostate cancer cell, subsequently activating downstream nuclear factor of activated T cells (NFAT) to promote the proliferation rate, and proliferating cell nuclear antigen (PCNA) expression [13]. However, our current study found that TRPV6 is frequently down-regulated in ESCC. Downregulation of TRPV6 in mRNA level was detected in 32 of 45 (71.1 %) of ESCC tumors and in 118 of 244 (48.4 %) informative ESCC tissues in protein level when compared with paired adjacent nontumor tissues. Besides, TRPV6 is down-regulated in ten ESCC cell lines when compared with pooled samples from 45 nontumor tissues (Np). Our result is consistent with a previous study by Wu et al.[26] which the expression of TRPV6 at protein and mRNA levels is markedly decreased in renal cell carcinoma than that in normal kidney tissue. Although the study only included twenty seven patients with renal cell carcinoma and did not detect the expression of TRPV6 in renal cell lines, the findings lend support to our claim that TRPV6 could be down-regulated in some cancer types. Thus, these findings suggested that expression of TRPV6 in cancers could be tissue-specific and lend strong rationale to explore its expression pattern in different cancer types. By far, this is the first comprehensive study to report that TRPV6 is down-regulated in ESCC. Furthermore, our present study showed that patients with downregulation of TRPV6 trend to have a higher rate of advanced pT stage in both mRNA cohort and protein cohort, but the result is not statistically significant. Our findings suggested that TRPV6 might have tumor-suppressive ability and inhibit the progression of ESCC. Previous studies have indicated that TRPV6 might play a protective role in some cancers [17, 18]. For instance, one study showed that TRPV6 can mediate capsaicin-induced apoptosis in gastric cells [18]. TRPV6 seems to display differing roles in the progression and proliferation of cancer dependent on cellular context. Thereby, further studies in vitro *and* in vivo are ongoing to clarify its tumor-suppressive ability and the precise mechanisms in ESCC.

The potential of TRPV6 as a marker to predict the clinical outcome of cancers has been well-established [9, 14, 27]. Substantial expression of TRPV6 mRNA increases with the degree of aggressiveness of the cancer and the degree of metastasis outside the prostate in patients with prostate cancer [9, 27]. Additionally, breast cancer patients with high expression of TRPV6 have a worse survival when compared to those with low or intermediate TRPV6 expression [14]. Although patients with downregulation of TRPV6 trend to have a poorer 3-year DSS, no significant association is observed between TRPV6 expression and survival of ESCC patients. Interestingly, stratified survival analysis based on the gender in two cohorts showed that downregulation of TRPV6 is significantly associated with an unfavorable 3-year DSS in male patients, while downregulation of TRPV6 is significantly related with favorable 3-year DSS in female patients. This result is the first time to suggest that TRPV6 plays a Janus-like role in predicting survival of male and female patients with ESCC. Epidemiological studies have revealed that male patients has a worse prognosis than female patients with esophageal cancer [27, 28]. More attentions should be paid to focusing on prognostic predictor for tailoring more effective therapies in male patients with ESCC. Our result found that male patients with downregulation of TRPV6 have a worse survival than those with normal level of TRPV6. This finding exhibits important clinical significance in determining TRPV6 expression status and identifying patients subpopulation at high risk of cancer-specific mortality, indicating that male patients with downregulation of TRPV6 are likely to benefit from adjuvant treatment.

Previous studies indicated that the expression of TRPV6 is directly or indirectly regulated by sex steroid hormones [8, 29–31]. TRPV6 was found to be negatively regulated by androgen in prostate cancer cell line [29]. In the presence of the specific androgen receptor antagonist, Casodex, TRPV6 mRNA level increases 2-fold over 2 days in a time-dependent manner. Meanwhile, addition of dihydrotestosterone (DHT) reduces TRPV6 mRNA level 80 % within one day. On the other hand, the expression of TRPV6 is up-regulated in time-dependent manner by estradiol and progesterone in breast cancer cell line [8]. These observations shed lights on why TRPV6 plays a mixed role in predicting survival of male and female patients with ESCC. However, further studies are needed to clarify the precise mechanisms.

We acknowledged that our study suffered from several limitations. To better elucidate the role of TRPV6 in cancers, the following challenges should be met in the future. First of all, our cohort study was retrospective study, which may lead to selection bias. Prospective studies are required to confirm its prognostic significance. Second, although this is the first study reporting TRPV6 is down-regulated in ESCC, the role of TRPV6 in proliferation and invasion of ESCC in vitro *and vivo* are yet to be elucidated. Further researches for cell cycle analysis, apoptosis analysis, invasion assays and tumor formation in vivo are required to explore the tumor-suppressive ability of TRPV6 and its related pathway. Third, the mechanism by which TRPV6 imposes opposite on survival of male and female patients with ESCC was not investigated in our study. Further researches about the plausible interaction between TRPV6 and sex hormones in ESCC are needed to unveil the precise mechanism.

## Conclusions

In summary, for the first time, we clarified that the expression of TRPV6 in mRNA and protein level was down-regulated in ESCC tissues. Analysis of two independent cohorts suggested that as a predictive biomarker, TRPV6 plays a Janus-like role in predicting survival of male and female patients.

## Abbreviations

CT: computed tomography
DHT: dihydrotestosterone
DSS: disease-specific survival
ESCC: esophageal squamous cell carcinoma
IHC: immunohistochemical
qRT-PCR: Quantitative Real-time Polymerase Chain Reaction
ROC: Receiver operative characteristic
TMA: tissue microarray
TRP: Transient receptor potential
TRPV6: transient receptor potential vanilloid 6
